# Spatial and seasonal influences on culturable endophytic mycobiota associated with different tissues of *Eugenia jambolana* Lam. and their antibacterial activity against MDR strains

**DOI:** 10.1186/s12866-016-0664-0

**Published:** 2016-03-18

**Authors:** Manila Yadav, Amita Yadav, Sandeep Kumar, Jaya Parkash Yadav

**Affiliations:** Department of Genetics, M. D. University, Rohtak, 124001 Haryana India

**Keywords:** *Eugenia jambolana*, Season, Sites, Tissues, Diversity, Endophytic fungi, Antibacterial, MDR

## Abstract

**Background:**

Present study focuses on diversity and distribution analysis of endophytic fungi associated with different tissues of *Eugenia jambolana*. The influence of season and geographical location on diversity and distribution of endophytic fungi has been analyzed. Antibacterial activity of isolated fungal species has also been investigated against MDR bacterial strains.

**Result:**

A total of 1896 endophytic fungal isolates were obtained from healthy, surface sterilized tissues of leaf, stem and petiole tissues during summer, monsoon and winter season. Out of 24 fungal species isolated, 20 species belong to class Ascomycetes, 2 to Basidiomycetes and 2 to Zygomycetes. Maximum species diversity was in rainy season whereas colonization frequency was in winter. All the diversity indices showed maximum species diversity at site 5 (Yamunanager), rainy among the seasons and leaf among the tissues studied. *Aspergillus* genus was most frequently isolated. *Aspergillus niger* and *Alternaria alternata* were most dominant species. Three way ANOVA results showed that effect of season was highly significant on species diversity in relation to sites and tissues. 60 % endophytic fungal extracts showed significant antibacterial activity against one or more than one MDR bacterial strain.

**Conclusion:**

Different fungal species were recovered from different sites but the inter-site comparisons were not significant according to Jaccard similarity coefficient. Diversity of such fungal endophytes indicates that *Eugenia jambolana* plant acts as an ecosystem facilitating survival of many microbes with impressive antibacterial potential.

**Electronic supplementary material:**

The online version of this article (doi:10.1186/s12866-016-0664-0) contains supplementary material, which is available to authorized users.

## Background

*Eugenia jambolana* (*Schizium cumini*) commonly known as Jamun or black plum is a popular plant in various traditional medicinal systems. It is well known cultivated tree of family Myrtaceae having large canopy. The plant is endogenous to Indian subcontinent, ranging from upper sub-Himalayan region to extreme southern region. Before the discovery of Insulin, Jamun was an integral part of alternative medicine system for the treatment of diabetes [1]. Different parts of Jamun tree have been reported to have antioxidant, anti-inflammatory, anti-microbial, antiviral, anti-diarrheal, antifertility, anorexigenic, gastroprotective, anti ulcerogenic, behavioural effects, radioprotective, hypoglycemic and antidiabetic activities [2].

In biosphere each macroscopic life is supported by large community of microscopic life hidden underneath [3]. Microbes that colonize the internal plant tissues without causing any apparent harm to their host plant are termed as endophytes [4, 5]. They spend the whole or at least a part of their life cycle, inter or intracellular inside the host plant tissue. Endophyte-host plant relation ranges from mutualism to antagonism or slightly pathogenesis [6, 7]. Endophytic microorganisms include fungi [8, 9], bacteria [10], actinomycetes [11, 12] and algae [13]. Fungi are the most frequently encountered endophytic microbes. Symbiotic association of fungi with photosynthetic organisms is universal and about 400 million years old [14]. Endophytic fungi are ecologically important components as they influence the diversity structure and dynamics of plant community [7]. Biology and ecology of these fungal endophytes are not well understood but are likely to change according to host and its environmental conditions [15]. These are beneficial for the host as they modulate its nutrition, metabolite production and stress response [16, 17]. A vast variety of compounds with proven pharmaceutical effects such as taxol [18], camptothecin [19], javanicin [20], azadiractin [21], ergoflavin [22], griseofulvin, hypericin [23] have been isolated from endophytic fungi. These compounds are produced due to different niches occupied by the endophytes [8, 24]. Sometimes similar compounds of host plant may be synthesized by the endophytic partner. The production of these similar secondary metabolites is a result of co-evolution and activation of diverse metabolic pathways to survive inside the host tissues [25, 26]. Therefore, endophytes are considered metabolically more active than free living microbes [27].

This study represents the first comprehensive step to investigate the species composition and distribution of culturable endophytic fungi within the internal tissues of *Eugenia jambolana*. The aim of present investigation was to evaluate the geographical, seasonal and tissue specific diversity with colonization frequency of endophytic mycoflora in *Eugenia jambolana*. In addition, inhibitory activity of these fungi against MDR (Multi Drug Resistant) bacterial strains was also examined.

## Results

### Mycology

Total 2430 segments from three tissues viz. leaf; stem and petiole were used for endophytic fungal isolation. A total of 1896 (78 %) culturable endophytic fungal isolates were obtained, representing 14 genera and 24 species. 3 uncultured fungal sterile mycelia were also obtained. Microscopic images of endophytic fungi shows in (Additional file 1: Figure S1). Out of 24 fungal species isolated, 20 species belong to class Ascomycetes, 2 to Basidiomycetes and 2 to Zygomycetes. *Rhizoctonia solani* and *Coprinopsis cinerea* were the basidiomycetes fungi. Zygomycetes fungi isolated were *S. racemosum* and *Choanephora infundibulifera*. Species belonging to *Aspergillus* genus were most frequently isolated. *Aspergillus niger* and *Alternaria alternata* were most dominant species where as *Chaetomium globosum, Aspergillus japonicus*, *Aspergillus niger* strain, *Aspergillus aff. fumigatus* strain were very rare species in this study. *Rhizoctonia solani*, *Curvularia lunata* and *Alternaria alternata* were three dark septate fungi isolated in our study.

### Molecular identification

**Fig. 1:**
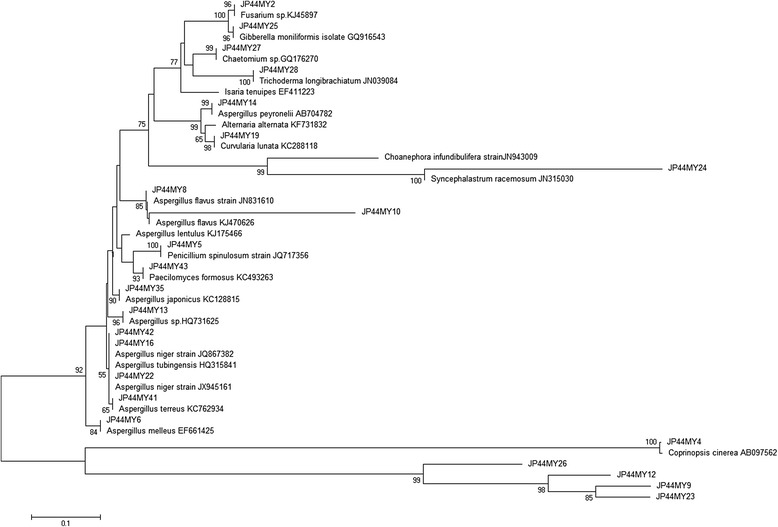
Phylogenic analysis of endophytic fungi isolated from the leaf, stem and petiole of *E. jambolana.* The phylogenic tree was constructed using Neighbor- Joining method. Bootstrap values above 50 % from 1000 replicates are indicated at each node

Sequences obtained after molecular characterization were submitted to NCBI GenBank database. Isolate JP44MY10 showed 78 % sequence similarity to *Fusarium solani* isolate. Another isolate JP44MY24 showed 93 % sequence similarity to *S. racemosum*. These 2 fungal isolates were designated as unidentified fungal strains. Rest of the sequences showed either 99 or 100 % sequence similarity on BLAST search. Accession number, best matched species and % similarity of all fungal isolates are given in Table 1. Phylogenetic tree was constructed on the basis of these sequences and the best matched sequences obtained from NCBI database (Fig. 1).

**Table 1:** List of endophytes isolated from *E. jambolana* and their Genbank accession no. with most closely related organisms

Fungal Isolates	Accession No.	Closest related species	Similarity
JP44MY2	KF031018	*Fusarium sp.*KC341961	99 %
JP44MY4	KF049006	*Coprinopsis cinerea genus* AB097562	99 %
JP44MY5	KF031019	*Penicillium spinulosum JQ*717356	99 %
JP44MY6	KF031020	*Aspergillus melleus* EF661425	100 %
JP44MY8	KF031021	*Aspergillus flavus JN831610*	99 %
JP44MY9	KF830207	*Aspergillus aff. fumigatus*	99 %
JP44MY10	KF031022	*Fusarium solani KC142125*	78 %
JP44MY12	KF830209	*Isaria tenuipes*	100 %
JP44MY13	KF031023	*Aspergillus sp.HQ731625*	100 %
JP44MY14	KF031024	*Aspergillus peyronelii EF669715*	99 %
JP44MY16	KF031027	*Aspergillus nigerJQ867382*	100 %
JP44MY19	KF031025	*Aspergillus tubingensis JN585941*	100 %
JP44MY22	KF031026	*Curlvularia lunata*	100 %
JP44MY23	KF830208	*Alternaria alternata*	100 %
JP44MY24	KJ136028	*Syncephalastrum racemosum JN315030*	92 %
JP44MY25	KF031028	*Gibberella moniliformisGQ916543*	99 %
JP44MY26	KF830210	*Choanephora infundibulifera*	98 %
JP44MY27	KF031029	*Chaetomium globosumJQ176270*	99 %
JP44MY28	KF031030	*Trichoderma longibrachiatum JNO39084*	99 %
JP44MY35	KF031031	*Aspergillus japonicas KC1288815*	100 %
JP44MY41	KF031032	*Aspergillus terreus KC113303*	100 %
JP44MY42	KF031033	*Aspergillus nigerJX945161*	100 %
JP44MY43	KF031034	*Pacilomyces formosus KC493264*	100 %

### Seasonal influence

Seasonal effect was significant on endophytic fungal diversity. Maximum Species diversity was in rainy season (24 species) and minimum in winter (17species). However, fungal species diversity data showed no correlation with number of isolates according to season. Maximum no. of isolates were obtained in winter season (762) followed by rainy (635) and then summer (499). Colonization frequency showed opposite trend to that of species diversity. It was maximum in winter and minimum in summer season (Tables 2, 3 and 4). All diversity indices confirmed seasonal effect on species diversity. Gleason index (4.02), Simpson index (0.91) and Shannon-Wiener diversity index (2.87) showed highest values for rainy season (Table 5). Result of Three way ANOVA showed that effect of season was highly significant on species diversity in relation to sites (*p* = 0.001) and tissues (*p* = 0.015) (Table 6). Some endophytic fungal species showed seasonal specificity. *Choanephora infundibulifera* was observed only in winter season*. Chaetomium globosum* and *Aspergillus terreus* were found both in winter and rainy season but not in summer season.

**Table 2:** Percent (CF) Colonization frequency of endophytic fungi in leaf, stem and petiole tissues of *E. jambolana* in Winter season

Endophytic fungi	Winter season																		
	Site 1			Site 2			Site 3			Site 4			Site 5			Site 6			Total
	L	S	P	L	S	P	L	S	P	L	S	P	L	S	P	L	S	P	
JP44MY2	1.4	–	–	–	–	–	.74	.74	–	2.2	2.2	.74	6.6	–	–	3.7	2.9	–	21.2
JP44MY3	5.1	13.3	–	–	7.4	–	5.9	10.3	.74	5.9	6.6	.74	–	7.4	–	2.2	1.4	–	66.9
JP44MY4	.74	.74	–	–	–	–	–	–	–	7.4	1.4	1.4	2.9	1.4	.74	–	.74	–	17.4
JP44MY5	.74	–	–	–	–	–	–	–	–	–	–	–	.74	–	.74	–	–	–	2.2
JP44MY6	2.9	.74	2.2	2.2	.74	–	7.4	1.4	–	11.1	1.4	1.4	1.4	–	–	2.9	–	–	35.7
JP44MY8	2.2	2.9	2.2	39.2	8.1	13.3	10.3	4.4	.74	2.9	2.9	.74	8.1	2.2	.74	6.6	1.4	.74	109.6
JP44MY9	–	–	–	–	–	–	–	–	–	–	–	–	–	–	–	–	–	–	–
JP44MY10	1.4	–	–	–	–	–	–	–	–	–	–	–	.74	–	–	–	–	–	2.2
JP44MY12	2.2	–	2.2	5.1	1.4	2.2	2.9	.74	–	–	–	–	–	–	–	–	–	–	16.7
JP44MY13	1.4	–	–	.74	–	–	–	–	–	2.9	–	–	.74	–	–	–	2.9	–	8.6
JP44MY14	8.8	2.2	2.2	–	.74	–	1.4	–	13.3	3.7	.74	–	24.4	.74	–	6.6	4.4	.74	69.9
JP44MY16	15.5	20	5.9	45.9	29.6	5.9	59.2	14.8	–	–	1.4	.74	8.8	4.4	1.4	9.6	–	.74	223.8
JP44MY18	3.7	–	–	–	–	–	–	–	–	12.5	1.4	–	4.4	.74	–	–	–	2.2	24.9
JP44MY22	–	–	–	–	–	–	–	–	–	1.4	.74	10.3	–	–	–	–	–	–	12.4
JP44MY23	5.9	2.9	–	5.1	–	–	7.4	2.2	–	49.6	13.3	–	21.4	5.1	1.4	40.7	14.0	2.9	171.9
JP44MY24	4.4	4.4	–	1.4	–	–	1.4	2.9	–	–	2.9	–	1.4	–	.74	–	.74	–	20.2
JP44MY25	–	–	–	–	–	–	–	–	–	–	–	–	–	–	–	–	–	–	–
JP44MY26	–	–	–	–	–	–	–	–	–	–	–	–	–	–	–	–	–	–	–
JP44MY27	–	–	–	–	–	–	–	–	–	–	–	–	–	–	–	–	–	–	–
JP44MY28	–	–	–	–	–	–	–	–	–	–	–	–	1.4	1.4	.74	–	–	–	3.5
JP44MY35	–	–	–	–	–	–	–	–	–	–	–	–	–	–	–	–	–	–	–
JP44MY41	2.2	4.4	6.6	–	–	1.4	8.8	4.4	–	–	–	–	–	–	–	2.9	.74	–	31.4
JP44MY42	–	–	–	–	–	–	–	–	–	–	–	–	–	–	–	–	–	–	–
JP44MY43	–	–	–	–	–	–	–	–	–	–	–	–	–	–	–	–	–	–	–
Total	59.9	51.5	21.3	99.6	48.9	22.8	105.5	41.8	14.7	99.6	35.0	16.0	83.0	23.4	6.5	75.2	29.2	7.3	

*L* Leaf tissue, *S* Stem tissue, *P* Petiole tissue

**Table 3:** Percent (CF) Colonization frequency of endophytic fungi in leaf, stem and petiole tissues of *E. jambolana* in Summer season

Endophytic fungi	Summer season																		
	Site 1			Site 2			Site 3			Site 4			Site 5			Site 6			Total
	L	S	P	L	S	P	L	S	P	L	S	P	L	S	P	L	S	P	
JP44MY2	–	–	–	–	–	–	–	–	–	–	–	–	.74	–	–	–	–	–	.74
JP44MY3	–	.74	–	–	2.2	–	–	5.1	–	–	.74	–	–	4.4	–	–	.74	–	13.9
JP44MY4	7.4	–	–	–	–	–	2.2	–	–	–	–	–	–	–	–	–	–	–	9.6
JP44MY5	–	–	–	–	.74	–	–	–	–	.74	–	–	–	–	–	–	–	–	1.4
JP44MY6	1.4	–	.74	2.2	–	–	2.9	1.4	–	1.4	.74	–	.74	.74	–	.74	1.4	–	14.4
JP44MY8	2.2	–	.74	.74	1.4	–	1.4	.74	–	2.2	.74	–	–	–	–	2.2	–	–	12.3
JP44MY9	–	–	–	.74	.74	–	–	–	–	–	–	–	–	–	–	–	–	–	1.4
JP44MY10	.74	.74	–	.74	–	–	2.2	1.4	–	2.2	.74	–	–	–	–	1.4	.74	–	10.9
JP44MY12	–	3.7	–	2.2	–	–	4.4	2.2	.74	–	1.4	–	.74	–	–	.74	–	–	16.12
JP44MY13	–	–	.74	–	–	–	–	–	–	–	–	–	–	–	–	–	–	–	.74
JP44MY14	4.4	2.2	–	5.1	3.7	–	11.8	4.4	–	–	.74	.74	.74	.74	–	1.4	–	–	35.9
JP44MY16	5.1	–	–	10.3	1.4	.74	8.1	2.2	–	–	6.6	.74	7.4	1.4	2.2	12.5	–	2.2	60.8
JP44MY18	2.9	.74	–	3.7	.74	–	1.4	1.4	–	2.2	.74	–	1.4	–	–	8.8	1.4	1.4	26.8
JP44MY22	1.4	.74	.74	.74	2.9	1.4	2.9	2.2	–	11.8	2.9	.74	–	–	–	.74	–	–	29.2
JP44MY23	18.5	5.1	8.1	13.3	2.9	–	46.6	21.4	8.1	5.9	–	–	1.4	–	–	3.7	1.4	.74	137.1
JP44MY24	–	–	–	1.4	–	–	–	–	–	–	–	–	–	.74	–	–	–	–	2.1
JP44MY25	–	–	–	1.4	–	–	1.4	–	–	2.2	–	–	–	–	–	–	–	–	5.0
JP44MY26	.74	–	–	–	–	–	–	–	–	–	–	–	.74	–	–	–	–	–	1.4
JP44MY27	–	–	–	–	–	–	–	–	–	–	–	–	–	–	–	–	–	–	–
JP44MY28	–	–	–	–	–	–	–	–	–	–	–	–	–	–	–	–	–	–	–
JP44MY35	.74	–	.74	–	.74	–	–	–	–	–	–	–	–	.74	–	–	–	–	2.9
JP44MY41	1.4	–	–	–	–	–	.74	–	–	–	–	–	2.2	–	–	–	–	–	4.3
JP44MY42	1.4	–	–	.74	–	–	–	–	–	2.2	–	–	–	–	–	.74	–	–	5.0
JP44MY43	–	–	–	–	–	–	–	–	–	–	–	–	.74	–	–	–	–	–	.74
Total	47.2	13.9	11.8	42.5	17.4	2.1	86.1	42.4	8.8	28.6	15.3	2.2	16.8	8.7	2.2	32.2	5.6	4.3	

*L* Leaf tissue, *S* Stem tissue, *P* Petiole tissue

**Table 4:** Percent (CF) Colonization frequency of endophytic fungi in leaf, stem and petiole tissues of *E. jambolana* in Rainy season

Endophytic fungi	Rainy season																		
	Site1			Site2			Site3			Site4			Site5			Site6			Total
	L	S	P	L	S	P	L	S	P	L	S	P	L	S	P	L	S	P	
JP44MY2	1.4	–	–	–	–	–	.74	–	.74	–	.74	–	3.7	–	1.4	3.7	–	–	12.4
JP44MY3	–	7.4	–	3.7	8.1	1.4	2.2	5.9	–	–	–	–	2.2	2.9	.74	–	11.1	2.2	47.8
JP44MY4	–	–	–	2.2	.74	–	.74	2.2	–	–	–	–	1.4	–	–	1.4	1.4	–	10.0
JP44MY5	.74	–	–	1.2	–	–	2.2	–	–	–	–	–	1.4	9.6	.74	–	–	–	15.8
JP44MY6	.74	2.2	–	2.2	.74	.74	1.4	–	–	.74	–	.74	1.4	–	–	3.7	5.1	–	19.7
JP44MY8	–	–	1.4	1.4	–	–	2.2	.74	–	–	–	.74	3.7	–	–	1.4	1.4	–	12.9
JP44MY9	1.4	–	–	–	–	–	.74	–	–	–	–	–	.74	–	–	–	–	–	2.9
JP44MY10	1.4	1.4	–	–	–	.74	.74	–	–	–	–	–	1.4	–	–	–	–	–	5.6
JP44MY12	2.2	2.9	–	3.7	1.4	1.4	2.2	2.2	–	–	–	–	1.4	1.4	–	1.4	.74	–	20.9
JP44MY13	–	–	1.4	1.4	–	–	2.9	–	–	1.4	.74	–	1.4	–	–	–	–	–	9.2
JP44MY14	9.6	4.4	2.2	5.1	1.4		28.8	.74	–	8.1	–	–	–	–	–	6.6	2.2	.74	69.8
JP44MY16	2.2	4.4	3.7	3.7	1.4	.74	8.8	12.5	7.4	11.1	–	.74	.74	.74	.74	7.4	–	–	66.3
JP44MY18	3.7	3.7	1.4	4.4	1.4	1.4	2.2	1.4	2.2	3.7	2.2	2.2	7.4	1.4	–	5.9	1.4	–	46.0
JP44MY22	1.4	–	–	.74	2.2	–	–	–	–	14.8	–	3.7	3.7	1.4	–	2.5	3.7	2.2	36.3
JP44MY23	5.1	–	.74	10.3	3.7	3.7	–	–	–	9.6	.74	.74	1.4	–	.74	.74	2.2	–	39.7
JP44MY24	–	.74	–	2.2	–	–	–	–	–	.74	–	–	–	–	–	–	–	–	3.6
JP44MY25	–	–	–	1.4	–	–	–	–	–	1.4	2.9	–	–	–	2.2	1.4	2.2	–	11.5
JP44MY26	–	–	–		–	–	–	–	–	–	–	–	.74	–	–	–	–	–	.74
JP44MY27	1.4	.74	1.4	.74	–	.74	–	–	–	–	.74	–	–	–	–	–	–	–	5.7
JP44MY28	–	–	–	–	–	–	–	–	–	–	.74	–	1.4	2.9	–	–	–	–	5.0
JP44MY35	.74	–	–	–	1.4	–	–	–	–	–	–	–	.74	–	–	–	.74	–	3.6
JP44MY41	–	–	–	.74	–	–	–	–	–	–	2.9	–	2.2	–	–	–	–	–	5.8
JP44MY42	2.9	–	.74	–	–	–	–	–	–	–	–	–	1.4	–	–	–	–	–	5.0
JP44MY43	–	–	–	.74	–	–	–	–	–	–	–	–	–	–	–	1.4	–	–	2.1
Total	34.9	27.9	12.9	45.8	22.4	10.8	55.	25.6	10.3	51.5	11.7	8.8	38.0	20.3	6.5	37.5	32.1	5.1	

*L* Leaf tissue, *S* Stem tissue, *P* Petiole tissue

**Table 5:** Species diversity in terms of richness, evenness and dominance of endophytic fungi in different seasons, sites and tissues as calculated by various diversity indices

Season/site/tissues	S	N_P_	G	GR	D	1-D	H’	E
Summer	499	22	3.219	0.040	0.110	0.890	2.545	0.82
Rainy	635	24	4.029	0.041	0.090	0.910	2.870	0.88
Winter	762	17	2.411	0.021	0.121	0.879	2.310	0.81
Site1	341	18	3.086	0.052	0.116	0.883	2.317	0.80
Site2	320	22	3.640	0.065	0.117	0.883	2.358	0.83
Site3	366	17	2.710	0.043	0.115	0.885	1.956	0.63
Site4	277	19	3.378	0.068	0.128	0.872	2.365	0.80
Site5	272	24	4.103	0.084	0.070	0.930	2.798	0.88
Site6	318	20	3.123	0.056	0.104	0.896	2.506	0.83
Stem	554	23	3.399	0.041	0.108	0.892	2.531	0.77
Petiole	177	18	3.091	0.020	0.149	0.851	2.239	0.77
Leaf	1165	24	3.640	0.090	0.096	0.904	2.618	0.80

*S* No. of isolates, *N*_*p*_ No. of species, *G* Gleason index, *GR* Relative index for Gleason index, *D* Simpson’s dominance ratio, *1-D* Simpson's Index of Diversity,

*H* ^*’*^ Shannon- Weiner index, *E* Pielou’s evenness ratio

**Table 6:** Three way ANOVA analysis of all variables

Source	df	MS	F values	% variance p
Season (1)	2	0.821	0.316	0.730 ns
Site (2)	5	2.717	1.046	0.395 ns
Tissue (3)	2	501.191	192.857	0.000***
1 × 2 × 3	20	3.630	1.397	0.14 ns
1 × 2	10	8.614	3.314	0.001***
1 × 3	4	8.404	3.234	0.015**
2 × 3	10	4.006	1.542	0.135 ns
Error	108	2.599		

***p > 0.001 = significant, ** p > 0.015=significant

*Ns* non significant

### Tissue specificity

**Fig. 2:**
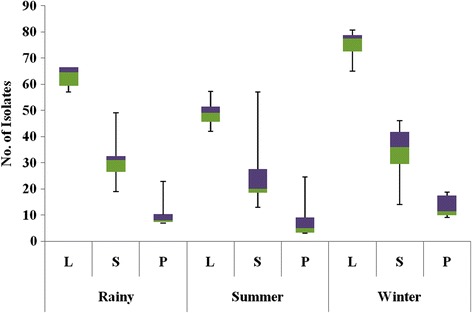
Boxplots depicting the effects of tissue in relation to season on colonization frequency of endophytic isolates

Species diversity was highly influenced by tissue types. All the 24 species detected were isolated from both leaves and stem. Only 18 species were recovered from petiole. Colonization frequency of endophytic fungi dominated leaf tissues, followed by stem and petiole (Tables 2, 3 and 4). Effect of tissue in relation to season on colonization frequency of endophytic isolates is shown in Fig. 2. Maximum 1135 isolates were recovered from leaf tissues. Value of all diversity indices like Gleason index (3.64), Simpson index (0.90) and Shannon-Wiener diversity index (2.61) was highest in leaf tissues. Three way ANOVA results verified that effect of leaf tissue on species diversity was highly significant with a *p* = 0.000 value (Table 6). *Aspergillus niger* strain was recovered only from leaf tissue.

### Effect of sites

Although various types of fungal species were recovered from different sites but the inter-site comparisons were not significant as calculated by Jaccard similarity coefficient (Table 7). Similarity of site 1 to site 4 and site 3 to site 6 was found to be a maximum of 76 % J_c_, while least J_c_ value of 51 % was between site 3 and site 5. Three way ANOVA results also indicated that site did not have any significant effect on species diversity (Table 6). Among all the 6 sites, site 5 had greatest species diversity of endophytic fungi. All the diversity indices showed maximum species diversity in site 5 (Table 5), which is relevant with colonization frequency data. Site 5 had the maximum value of Gleason index (4.1), Simpson index (0.93) and Shannon-Wiener diversity index (2.79). This site had maximum Pielou^’^s evenness index (0.88) as compared to other sites. The lowest diversity was in site 3 as shown by Gleason index (2.710), Simpson index (0.88) and Shannon-Wiener index (1.95).

**Table 7:** Jaccard similarity coefficient values showing % similarity of isolates between 1sampling sites

Sampling location	Site 1	Site 2	Site 3	Site 4	Site 5	Site 6
Site 1	100					
Site 2	66	100				
Site 3	75	69	100			
Site 4	76	57	71	100		
Site 5	62	70	51	63	100	
Site 6	65	75	76	62	69	100

### Antibacterial activity of endophytic fungi

Out of 10 tested endophytic fungal extracts only six (60 %) inhibited the growth of one or more than one MDR bacterial strain (Table 8). Two endophytic fungi i.e. *Aspergillus terreus* and *Aspergillus tubingensis* inhibited the growth of both *Klebsiella pneumoniae* and *Pseudomonas aeruginosa* MDR strains*.* Endophytic fungi *Aspergillus niger, Chaetomium globosum* and *Aspergillus flavus* repressed *Klebsiella pneumonia* strain. *Pseudomonas aeruginosa* was inhibited by *Aspergillus* species. The diameter of inhibition zone varied between 10 to16 mm (Additional file 1: Figure S2). None of the fungal extracts showed prominent activity against MDR strain of *E. coli*. MIC range of fungal extracts varies from 625 μg/ml to 2.50 mg/ml (Table 9). Fungal extracts which had maximum inhibition zone exhibited minimum MIC value. In our study endophytic fungal extracts of *Aspergillus niger* and *Aspergillus terreus* had least MIC range of 625 μg/ml against *Klebsiella pneumonia*.

**Table 8:** Antibacterial activity of endophytic fungi against MDR bacterial strains

Fungal Endophytes	Diameter of inhibition zone (mm)		
	KP	PA	EC
*Coprinopsis cinerea*	–	–	–
*Penicillium spinulosum*	–	–	–
*Aspergillus flavus*	11.0 ± 0.76	–	–
*Aspergillus* sp.	–	12.15 ± 0.28	–
*Aspergillus peyronelii*	–	–	–
*Aspergillus niger*	16.0 ± 0.5	–	–
*Aspergillus tubingensis*	14.5 ± 0.57	11.0 ± 0.12	–
*Chaetomium globosum*	14.6 ± 0.57	–	–
*Aspergillus terreus*	16.8 ± 0.50	11.00 ± 1.0	–
*P. formosus*	–	–	–
Control	22.0 ± 0.50	20.0 ± 0.50	18.0 ± 0.57

Bacterial strains: *KP Klebsiella pneumoniae*, *PA Pseudomonas aeruginosa, EC Escherichia coli*

**Table 9:** Minimum Inhibitory Concentration (MIC in mg/ml) of endophytic fungal extracts against MDR bacterial strains

Bacterial strains	*A. flavus*	*Aspergillus* sp*.*	*A. niger*	*A. tubingensis*	*C. globosum*	*A. terreus*
KP	2.50	–	.62	1.25	1.25	.62
PA	–	2.50	–	2.50	–	2.50

Bacterial strains: *KP Klebsiella pneumoniae*, *PA Pseudomonas aeruginosa*

## Discussion

**Fig. 3:**
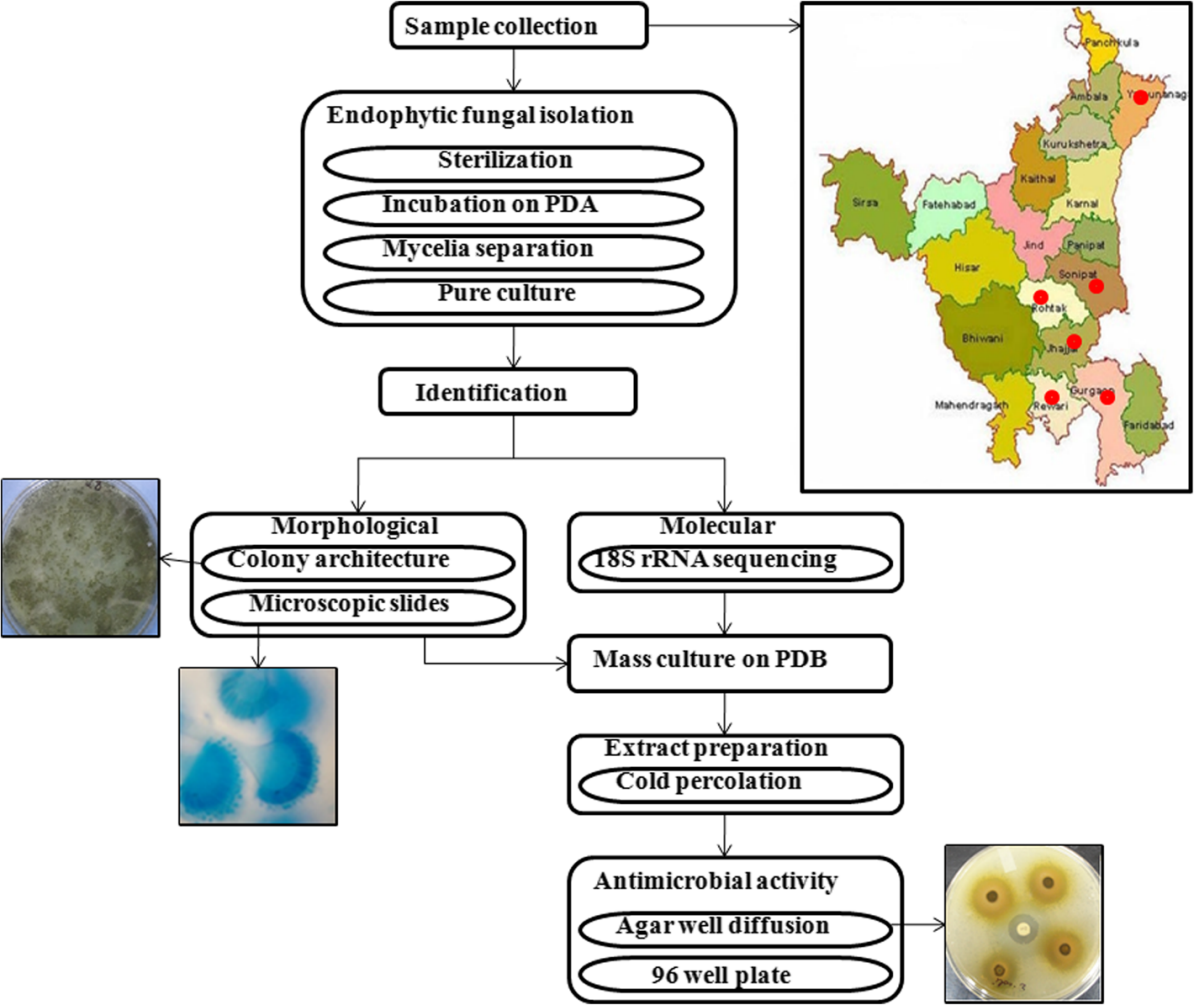
Schematic diagram of total work plan of study (Map URL http://haryana.gov.in/knowharyana/districts.html)

Endophytes are taxonomically diverse and species rich microbes. In this study 24 culturable isolated belonging to 14 genera and 3 divisions of fungi. Previous studies stated that class II endophytes infecting higher plants belong to either ascomycetes or basidiomycetes; ascomycetes and their anamorphs being the dominant endophytes [28, 29]. But recent studies showed that zygomycetes fungi also act as endophytes in aerial parts of higher plants [30, 31]. Our results link with both of the above cited findings. In our study 2 species belongs to zygomycota, 2 to basidiomycota and rest to ascomycota. Total work plan of the conducted study has been depicted in a brief schematic diagram (Fig. 3).

Generally, a consistent pattern is shown by foliar endophytes of a single host with some core dominant fungal species [32, 33]. Isolation of rare endophytic fungi depends on the methods of isolation and recovery of these species from the samples. If isolation methods are inefficient, similar core fungal species will be obtained from each site, no matter how distant they are [34, 35]. In our study *Aspergillus niger*, *Aspergillus flavus*, *Aspergillus peyronelii*, *Alternaria alternata* and *Rhizoctonia solani* constituted the core fungi. Four rare species were isolated from leaf and stem tissues. Our results demonstrate that the sampling method is quite satisfactory for rare fungal species isolation. Identification of fungal species was done using nuclear ribosomal internal transcribed spacer region. ITS regions are frequently used in fungal taxonomic studies due to high variability in this region of rDNA. This method is widely used as it provides detection of endophytes below the species level [36].

Tissue type has a prominent effect on species diversity and colonization frequency of endophytic fungal community. Some previous studies of endophytic fungi isolation on various Indian medicinal plants unveiled that colonization frequency and species recovery was highest in leaf tissues [24, 32, 37, 38]. Finding of our study corroborates with upper mentioned result of higher diversity in leaf tissue. *Eugenia jambolana* is a woody perennial tree with a very large canopy. Growth stage of tissue and location in the canopy affects the distribution of endophytes in perennial trees [32]. This can be a result of greater surface area of leaves that is exposed to outer environment. Moreover leaves have stomata that act as channel for entry of fungal mycelia [39, 40].

Seasons has eminent impact on recovery of endophytic population. Maximum fungal diversity is usually observed in rainy season as high humidity and temperature favours the endophytic fungal growth [32, 41] and also aids in dispersal of spores. Our study also confirmed that maximum diversity of species is found in rainy seasons. But maximum number of isolates was obtained in winter season which is in supported by the studies conducted on medicinal plants of Western Ghats in southern India by Neik et al. [31, 42]. These seasonal changes in species composition are due to different selection pressure on endophytes in different seasons inside plant tissues [43, 44].

Geographical locations affect the blueprint of endophytic population in medicinal plants. Probability of recovery of different endophytic fungal taxa increases in relation to distance between sites [45]. In our study maximum species recovery was from site 5, which is far distant from other site. However, intersite comparison showed no prominent effect on fungal diversity, which correlates with some earlier study on medicinal plants [32, 37]. Colonization frequency values vary widely according to sites. This variability is attributed to environmental conditions of the sites used in this study. Site 3 and site 6 are moderately polluted sites due to industrialization. SO_2_ and NO_2_ levels are high in these sites as compared to others [46–48]. High spore producing air borne fungi that act both as epiphytes and endophytes were more abundant in these sites. Sterilization procedure was effective to detect any of the epiphytes. Airborne fungi with high spore production are the most frequently isolated endophytes from many plants [49, 50]. In our study high spore producing cosmopolitan fungi *Alternaria alternata* and *Aspergillus niger* were most dominant endophytes. Different species of *Alternaria and Aspergillus* were dominant endophytes isolated in many studies conducted on tropical plants [51, 52].

Dark septate endophytic fungi mostly inhabit root tissues [53]. But in our study 3 dark septate endophytes viz. *Rhizoctonia solani*, *Curvularia lunata* and *Alternaria alternata* were frequently isolated from leaf and stem tissues, which proves that they are not confined to mycorrhizal tissues. Dark septate fungi help plant to survive against abiotic stress generated due to oxygen radicals [54]. They act as natural antioxidants of plant community. Plants inhabit a vast diversity of mycobiota which still need to be explored. In our study 2 unidentified fungal strains were discovered. Fungal isolates having 97 % or less rRNA region similarity are considered as different strains. 13.9 % strains of unknown genera have been isolated from plants of western Himalayas [8]. Taxonomic uniqueness of endophytes may result in novel chemistry thus paving the way for isolation of new metabolites. More than 8600 compounds of therapeutic, industrial and agricultural applications have been isolated from fungi and many more need to be explored [55].

This study is the first most comprehensive collection of endophytic fungi from multiple sites and seasons in different tissues of *Eugenia jambolana*. Information of endophytic fungal assemblage in plants could serve as a database reference for assessing fungal diversity from other geographical location [56]. Various diversity indices exhibited the diversity of fungal endophytic population with relation to above mentioned factors. Our study will help in understanding the ecology and community structure of endophytic fungal association in *Eugenia jambolana*.

The antibiotic resistance has become a global problem now a day. Research on more efficient antimicrobials has encouraged scientists to screen natural bioactive compounds from microbes. This study confirms that endophytic fungi isolated from *Eugenia jambolana* have effective antibacterial activity against MDR strains. Result of our previous studies on these endophytes proved their antimicrobial and antioxidant potential with a great amount of total phenols and alkaloids [57, 58]. These secondary metabolites mainly interfere with DNA replication and transcription processes as these intercalate between DNA double helix [59, 60]. Some of the reported antimicrobial alkaloids act by altering biosynthesis of bacterial cell wall [61]. Endophytic fungal species like *Penicillium sp., Aspergillus fumigatus, A. niger, Chaetomium globosum, Curvularia lunata* and *Fusarium* sp. isolated in our study have been previously reported for their antimicrobial potential. None of the endophytic fungal strain showed activity against *E. coli* bacteria. These results were consistent with the study of root endophytic fungi of *Panax ginseng* [62]. In present study most of endophytic fungal strains which displayed antibacterial activity belong to Aspergillus genus. Aspergillus genus is well studied taxa with more than 1200 reported biologically active secondary metabolites and their analogues [63]. Isolation of similar endophytes from *Eugenia jambolana* provides a platform to screen them as a new antimicrobial source.

## Conclusion

Nature is the largest repository of innumerable chemical structure with amazing bioactive prospective that cannot be recreated in the laboratories. Endophytic fungi are relatively less explored natural resource that can be a promising source of secondary metabolites. *Eugenia jambolana* hosts a great diversity of endophytic fungi. Various factors like tissue types seasons have noteworthy impact on diversity and colonization frequency of endophytic population as compared to geographical locations. Excellent antibacterial activity against MDR strains encourages us to design putative bioactive compounds from these endophytes.

## Methods

The protocol of the study was approved of Departmental Committee, Departmental Research Committee and P.G. Board of Studies of Genetics Department, M. D. University, Rohtak, India.

### Collection of plant sample

Plant material was collected from six different districts of Haryana (India), each having 3 sub-sites (Table 10). Distance between each sub-site was 30 Km. Samples were collected during summer, rainy and winter season from each site between months of December, 2010 to August 2011. Healthy leaves, stems and petioles of *Eugenia jambolana* (Voucher No. -64684, FRI Dehradun) were collected from individual plants at each location in triplicates. Tissues were placed in sterile plastic bags. All samples were brought to the laboratory in an ice box and processed further for endophytic fungi isolation within 48 h. Samples were collected from same plant in each season.

**Table 10:** Sample collection sites with average rainfall

Site	Name	Coordinates	Rainfall(mm)
1	Rohtak	28.89^°^ N 76.57^°^ E	458.5
2	Jhajjar	28.62^°^ N 76.65 E	444.0
3	Gurgaon	28.42^°^ N 77.03^°^ E	797.3
4	Rewari	28.18^°^ N 76.62^°^ E	553.0
5	Yamunanager	30.13^°^ N 77.28^°^ E	970.3
6	Sonepat	28.99^°^ N 77.02^°^ E	767.9

### Isolation of endophytic fungi

Plant samples were washed thoroughly in running tap water and rinsed with double-distilled water. All samples were surface sterilized by dipping in 70 % ethanol for 1 min, followed by 5 % sodium hypochlorite for 3 min and finally immersed in 75 % ethanol for 30 s [7, 34, 64]. Surface sterilized samples were rinsed in double distilled water twice and dried under aseptic conditions. The samples were cut into small pieces (0.5 x 0.5 cm) for leaves and 0.5 cm in length for stem and petiole. These samples were placed on petri dishes containing potato dextrose agar (PDA) supplemented with 150 mg/l streptomycin and sealed using Parafilm™. Efficiency of surface sterilization was tested by plating out 500 μl of the last rinsing water from the sterilization procedure and tissue imprint on fresh PDA plates [64]. Plates were incubated at 26 ± 1 °C until fungal growth was initiated. The growing tips of fungal mycelia were transferred to new PDA plates for pure culture. Pure cultures were examined periodically.

## Identification

### Morphological examination

The endophyte identification procedures were based on morphological structure of colony, fruiting body and arrangement of spores. Temporary mounts of the fungi were made in lacto phenol cotton blue. The fungi were identified using relevant keys and taxonomic notes [65].

### Molecular characterization and phylogenetic analyses of endophytic fungi

Fungal DNA isolation was performed using HiPura™ Fungal DNA Purification Kit (Himedia Laboratories) according to the kit user’s manual. Isolated DNA was stored at 4 °C until further use. PCR protocol was based on amplification of internal transcribed spacer (ITS) fragments (ITS1-5.8S-ITS2 rDNA) using the universal primers ITS1 (5’- TCCGTAGGTGAACCTGCGG -3’) and ITS4 (5’-TCCTCCGCTTATTGATATGC -3’) [66]. The reaction mixture contained 0.5 μl Taq Buffer, 0.4 μl dNTPS, 1.5 μl of each primer, 1.5 μl (5 unit) Taq DNA Polymerase and 2.5 μl (100 ng/μl) template DNA and PCR water was added to make a final volume of 50 μl. The PCR reaction was performed using following program: initial denaturation for 4 min at 94 °C, followed by 35 cycles of amplification (denaturation for 1 min at 94 °C, annealing for 30 s at 57 °C and extension for 1 min at 72 °C) and final extension of 7 min at 72 °C. The PCR products were electrophoresed in 1 % agarose gel for 30 min in TAE buffer. PCR amplicons were purified using Quick PCR Purification kit (Bangalore GENEI, INDIA). The purified products were sequenced using the facility of Genetix Biotech Asia Pvt. Ltd., India. Gene sequences were further compared with the NCBI GenBank database using BLASTN program (http://blast.ncbi.nlm.nih.gov/Blast.cgi). Sequences with more than 97 % similarity were considered as same strain [67, 68]. All sequences were submitted to GenBank database.

### Statistical analyses

Fungal growth measurement was calculated in terms of colonization frequency (CF) as given by Fisher & Petrini (1987) [69] -

$$ \mathrm{Colonization}\ \mathrm{frequency}\kern0.5em =\frac{\mathrm{No}.\ \mathrm{of}\ \mathrm{segments}\ \mathrm{colonized}\ \mathrm{b}\mathrm{y}\ \mathrm{a}\ \mathrm{single}\ \mathrm{endophyte}}{\mathrm{Total}\ \mathrm{no}.\ \mathrm{of}\ \mathrm{segments}\ \mathrm{incubated}}\times 100 $$

Species diversity was calculated by various diversity indices. Gleason and Shannon-Wiener indices represent richness aspect of diversity. Relative index of Gleason shows ratio of species richness over evenness. Simpson index (D), Simpson diversity index (1-D) is a measure of dominance of 1 or 2 fungal species. Pielou’s evenness index represents the evenness of isolated fungal species. To determine the effect of location, tissue type and season, the data containing no. of species isolates was subjected to three way ANOVA using STATISTICA 7. Jaccard similarity coefficient was determined for the locations chosen for the study with the help of NTSys software PC version 2.02e. Box plot was constructed to show the effect of season and tissue on no. of isolates. All sequences obtained in this study and their best matched sequences were subjected to multiple sequence alignment using Clustal W. Phylogenetic tree was constructed using MEGA 6 by Neighbour Joining method with 1000 bootstraps. Evolutionary distances were calculated using the Maximum Composite Likelyhood method [70]. All positions containing gaps and missing data were eliminated.

## Antibacterial activity

### Preparation of crude fungal extracts

The endophytes were mass cultured on Potato Dextrose Broth (PDB) media for 7–10 days at 27 °C in incubator shaker at 160 rpm. The mycelia were filtered and dried. The dried powdered materials were then extracted with organic solvent ethyl acetate (1:10) by cold percolation for 48–72 h. The obtained extract was then filtered using Whatman No. 1 filter paper and then concentrated under vacuum at 40 °C by using a rotary evaporator.

### Bacterial strains

Three multi drug resistant bacterial strains *Escherichia coli* (MDREC1)*, Klebsiella pneumonia* (MDRKP2)*, Pseudomonas aeruginosa* (MDRPA3) were obtained from the Microbiology Department of Post Graduate Institute of Medical Sciences, Rohtak, Haryana, India. The purity and identity of each isolate was confirmed in laboratory by standard microbiological methods [71–73].

### Agar well diffusion assay

The antibacterial activity of 10 selected endophytic fungi was tested by Agar well diffusion method [74]. A final stock concentration of 50 mg/ml was prepared from crude extracts. 10 μl, 20 μl, 30 μl, 40 μl of extract was poured to each well to determine the antibacterial effect. Streptomycin discs for bacteria (10 μg/disc) were used as positive controls. Diameter (mm) of the clear inhibition zone was measured to determine the antibacterial activity. Each experiment was done in triplicate and interpretation was based on average value of results.

### Minimum inhibitory concentration (MIC)

Minimum inhibitory concentration (MIC) is the lowest concentration of an antimicrobial that will inhibit the visible growth of a microorganism after overnight incubation. The MIC values of fungal extracts were determined based on a micro broth dilution method in 96 multi-well micro titer plates with slight modifications [75]. The plates were prepared in triplicate and incubated at 37 °C for 18 to 24 h. Color changes of resazurin indicator from purple to pink or to colorless indicated growth of microbes. The lowest concentration at which no color change occurred was taken as the MIC value of extract.

## Availability of data and materials

Phylogenic data has been deposited in Dryad (http://datadryad.org/). Provisional DOI is http://dx.doi.org/10.5061/dryad.3t758.

## Additional file

Additional file 1: Figure S1. Shows the details of microscopic image of endophytic fungi isolated from *Eugenia jambolana*. **Figure S2.** shows the details of antibacterial activity of endophytic fungal crude extracts against MDR bacterial strains. (DOC 2803 kb)
