# Supplementary material for: Spatial and seasonal influences on culturable endophytic mycobiota associated with different tissues of Eugenia jambolana Lam. and their antibacterial activity against MDR strains
Source: BMC Microbiol. 2016 Mar 18;16:44. doi: 10.1186/s12866-016-0664-0 (PMC4797120; doi:10.1186/s12866-016-0664-0)

File name: Additional Figure 1 (AF1)

Title of data: Microscopic image of endophytic fungi isolated from *Eugenia jambolana*.

Description of data:

1. *Fusarium sp.* 2. *Rhizoctonia solani* 3. *Coprinopsis cinerea* 4. *Penicillium spinulosum*

5. *Aspergillus melleus* 6. *Aspergillus flavus* 7. *Aspergillus aff. fumigatus* 8. *Fusarium solani*

9. *Isaria tenuipes* 10. *Aspergillus* sp. 11. *Aspergillus peyronelii* 12. *Aspergillus niger*

13. *Aspergillus tubingensis* 14. *Curlvularia lunata* 15. *Alternaria alternata* 16. *Syncephalastrum racemosum* 17. *Gibberella moniliformis* 18. *Choanephora infundibulifera* 19. *Chaetomium globosum* 20. *Trichoderma longibrachiatum* 21. *Aspergillus japonicas* 22. *Aspergillus terreus* 23. *Aspergillus niger* strain 24. *Pacilomyces formosus* 25. sterile mycelium


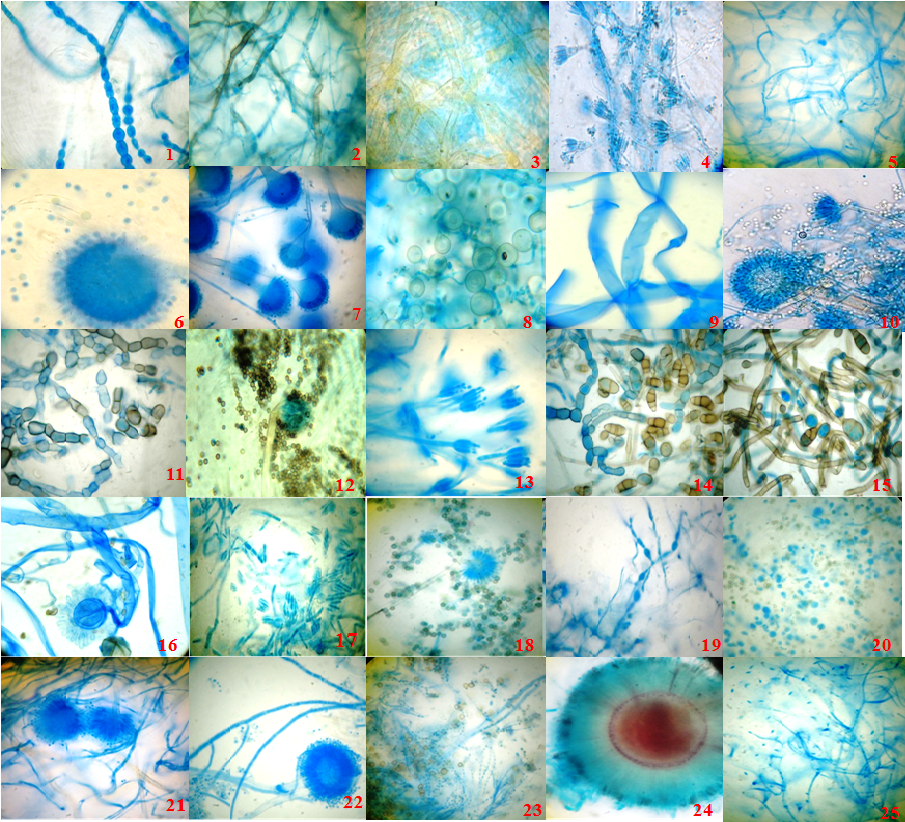


File name: Additional Figure 2 (AF2)

Title of data: Pictures showing antibacterial activity of endophytic fungal crude extracts against MDR bacterial strains.

Description of data:

1. Zone of inhibition of *Aspergillus peyronelii* extract against *E.coli* MDR.
2. Zone of inhibition of *Chaetomium globosum* extract against *Klebsiella pneumoniae* MDR.
3. Zone of inhibition of *Aspergillus* sp. extract against *Pseudomonas aeruginosa* MDR.
4. Zone of inhibition of *Aspergillus terreus* extract against *Klebsiella pneumoniae* MDR.
5. Zone of inhibition of *Aspergillus niger* extract against *Klebsiella pneumoniae* MDR.
6. Zone of inhibition of *Aspergillus terreus* extract against *Pseudomonas aeruginosa* MDR.
7. Zone of inhibition of *Aspergillus niger* extract against *E.coli* MDR.
8. Zone of inhibition of *Aspergillus tubingensis* extract against *Pseudomonas aeruginosa* MDR.


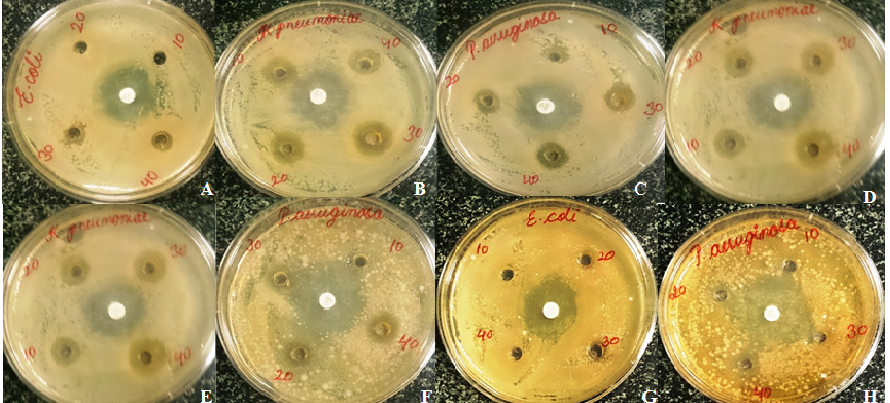

Supplement: Additional file 1: Figure S1. — Shows the details of microscopic image of endophytic fungi isolated from Eugenia jambolana. Figure S2. shows the details of antibacterial activity of endophytic fungal crude extracts against MDR bacterial strains. (DOC 2803 kb) [file 12866_2016_664_MOESM1_ESM.doc]
